# To Treat or to Extract Necrotic First Permanent Molars Between 8 and 12 Years of Age: A Retrospective Cohort Study

**DOI:** 10.3390/jcm13216596

**Published:** 2024-11-02

**Authors:** Valentin Vergier, Pierre-Jean Berat, Anne-Margaux Collignon, Sibylle Vital, Anne-Laure Bonnet

**Affiliations:** 1 Orofacial Pathologies, Imaging, and Biotherapies Laboratory (URP 2496 BRIO), Université Paris Cité, 92120 Montrouge, France; valentin.vergier@aphp.fr (V.V.); anne-margaux.collignon@aphp.fr (A.-M.C.); sibylle.vital@aphp.fr (S.V.); 2 Pediatric Dentistry, Service de Médecine Bucco-Dentaire, Département Médico-Universitaire CHIR, Hôpital Charles Foix, Assistance Publique des Hopitaux de Paris, Université Paris Cité, 94200 Ivry-sur-Seine, France; 3 Pediatric and Endodontic Dentistry, Service de Médecine Bucco-Dentaire, Département Médico-Universitaire ESPRIT, Hôpital Louis Mourier, Assistance Publique des Hopitaux de Paris, Université Paris Cité, 92700 Colombes, France; pierre.berat@aphp.fr; 4 Education and Health Promotion Laboratory (LEPS UR 3412), Unité de Formation et de Recherche Santé, Médecine et Biologie humaine, Université Sorbonne Paris-Nord, 93017 Bobigny, France

**Keywords:** pediatric dentistry, first permanent molar, extraction, endodontics, root canal treatment

## Abstract

**Background:** First permanent molars (FPM) are highly susceptible to decay before the age of 15. When they are severely affected, the decision between conservation and extraction arises, particularly considering the potential for the second permanent molar (SPM) to replace the FPM. This cohort study aimed to evaluate clinical practices regarding FPM pulp necrosis treatment in children aged eight to twelve in two hospital departments in the Paris region. A second objective was to evaluate the one-year outcomes of the two therapies. **Methods:** A retrospective analysis was conducted using computerized billing software to identify patients aged eight to twelve who underwent either extraction or root canal treatment (RCT). Data collected included sex, age, arch involved, number of decayed surfaces, presence of Molar Incisor Hypomineralization (MIH), presence of infection, and treatment type. **Results:** A total of 66 patients were included, representing 61 extracted teeth and 23 RCT. Three main decision criteria were identified: presence of MIH (*p* < 0.005), extent of decay (*p* < 0.05), and SPM Nolla’s stage. A total of 48% of the patients were seen at one year. A total of 16 of the 32 extractions and five of the nine RCTs had favorable evolution (*p* = 1). **Conclusions:** The question of whether to perform RCT or extraction of necrotic FPM in children aged eight to twelve is difficult to assess. It appears that five criteria need to be considered before the decision: possibility of long-term sealed coronal reconstruction, SPM Nolla’s stage, follow-up possibilities, arch concerned, and presence of third permanent molar.

## 1. Introduction

The first permanent molars (FPMs) erupt around the age of six, emerging behind primary teeth without replacing them. This eruption can go unnoticed if it is painless. During their maturation phase, these teeth are particularly difficult to clean, with the eruption process lasting 15 months, limiting natural self-cleaning [1]. Their posterior location further complicates parental supervision. FPM are known to be the most prone to decay before the age of 15, with more than 60% of children having an FPM decayed before the age of 13 [2,3]. Moreover, they may also suffer from structural defects such as Molar Incisor Hypomineralization (MIH), which affects 13.5% of children and increases their susceptibility to decay [4,5,6].

When FPMs are severely decayed or affected by structural defects, the question of whether to preserve them arises, especially when there is pulp involvement [3,7]. In cases where the pulp is vital, several therapies, such as cervical pulpotomy and pulp capping, can be successfully applied. However, this issue becomes more complex when assessing non-vital teeth, often requiring an endodontic specialist or specific sedation techniques [8].

Indeed, with the possibility of replacing the FPM by the second permanent molar (SPM), an alternative to preserving FPM “at all costs” can be considered. This raises the question of the decision-making process, which involves evaluating the likelihood of spontaneous SPM replacement for the FPM, along with the success rates of root canal treatments (RCT) and long-term restorations [9].

Extraction with spontaneous space closure is a well-established treatment, with a success rate ranging from 89.9% to 100% for maxillary FPM [10,11,12]. For mandibular FPM, this rate is lower, between 49% and 66% [10,12,13], because of the high density of the mandibular alveolar bone. These results are heterogeneous and the question of predicting space closure does not meet with consensus [14].

However, some authors have sought to identify criteria that could help predict space closure. Three major criteria were proposed:

- The SPM Nolla’s stage should be six, seven, or eight at the time of extraction, which typically occurs between eight and twelve [11,13];
- The presence of the third permanent molar (TPM) [10,13,15];
- For the mandible specifically, the mesial angulation of the SPM [10,13]. This criterion is evaluated using a radiograph, by tracing the SPM frontal axes of both FPM and SPM: if the axes cross at the coronal part, the angulation is mesial. If the axes are parallel, the angulation is orthogonal. If they cross at the apical part, the angulation is distal.

Endodontic treatment of immature necrotic teeth presents specific challenges related to biological, mechanical, infectious, and therapeutic factors. Immature permanent teeth with pulp necrosis, with or without apical periodontitis, pose particular technical difficulties for practitioners due to their incomplete root development, which makes conventional root canal treatment (RCT) unsuitable. In these cases, alternative approaches, such as the apical plug technique or revitalization procedures, may be necessary.

For mature permanent teeth in adults, traditional RCT remains the preferred treatment, offering a long-term success rate of over 86% after 10 years [16]. However, in adolescents, the success rate tends to decrease, with studies showing a success rate of approximately 79.6% [17].

In younger patients, particularly those under 10 years old with immature teeth, the success of regenerative endodontic treatments, such as pulp revascularization and revitalization, tends to be more variable. Reported success rates are approximately 50% in cases where pulp necrosis is caused by dental caries [18]. The success of these procedures can be significantly influenced by the practitioner’s expertise, collaboration with specialists, and the availability of advanced techniques and materials. In some cases, the success rate of RCT can drop as low as 36% after three years of follow-up, highlighting the impact of these external factors [19].

In absence of a consensus, the decision between these two treatments often relies on the clinician’s experience and the available facilities.

The objective of this cohort study was to evaluate the clinical practices of two hospital departments in Paris region (France) in managing FPM pulp necrosis in children aged eight to twelve. We aimed to investigate the criteria used to select a treatment when the FPM was severely damaged and necrotic.

A second objective was to evaluate the one-year outcomes of the two treatment options, extraction or RCT. This study aimed to provide a better understanding of the decision-making criteria that can guide dentists in choosing one treatment over the other.

## 2. Materials and Methods

### 2.1. Protocol

The protocol for this retrospective cohort study was developed following the STROBE checklist for cohort studies [20]. The study was designed to address the following question “what therapeutic decision criteria are considered after the diagnosis of FPM pulp necrosis in an eight- to twelve-year-old child in a hospital setting?”.

A secondary objective was to evaluate the success rate of the different treatments at one year of follow-up, in order to assess the relevance of these decision-making criteria.

The study was registered to the Assistance Publique des Hopitaux de Paris (AP-HP) medical data protection committee under the reference CFX-2023-R-CEPMP on 14 March 2023.

### 2.2. Patient Selection

The study was conducted in the Department of Pediatric Dentistry at Charles Foix Hospital (AP-HP, Ivry-sur-Seine, France) and Louis Mourier Hospital (AP-HP, Colombes, France).

Computerized billing software was used to identify all patients aged eight to twelve who underwent either an extraction or a RCT of one or more FPMs between 1 January 2019 and 31 January 2022. To identify all patients within this age range, those born between 1 January 2007 and 31 January 2014 were retrieved from the database. Patients outside this age range were then manually removed.

### 2.3. Inclusion and Exclusion Criteria

To be included, the patients had to be older than seven years and eleven months, the mean age when the SPM is at Nolla’s stage six [21]. This stage is the ideal time to consider spontaneous space closure following FPM extraction [10]. They also had to be younger than twelve years and two months, the mean age when the SPM is at Nolla’s stage eight [21]. This stage is the latest time to consider spontaneous space closure following FPM extraction [10]. Patients needed to have had a radiograph before treatment, either intra-oral or panoramic, to evaluate the FPM degradation and the SPM development stage.

Children with disabilities, for whom clinical and radiological examinations were not possible at the follow-up, patients who received vital pulp therapy instead of complete RCT (selection error), patients who had extractions due to internal or external resorption, and patients without radiographs before the treatment were not included in the study. Families were contacted up to five times, and a letter was sent to non-respondents. Patients who did not attend the one-year follow-up appointment were excluded from the secondary objective assessment.

### 2.4. Data Collection Process

For each patient, one author (V.V.) collected the following clinical data: sex, age at the time of treatment, affected arch (maxillary or mandibular), number of decayed surfaces, presence of MIH, presence of infection (Acute Apical periodontitis (AAP), abscess, cellulitis), and treatment (extraction or RCT).

**Figure 1:**
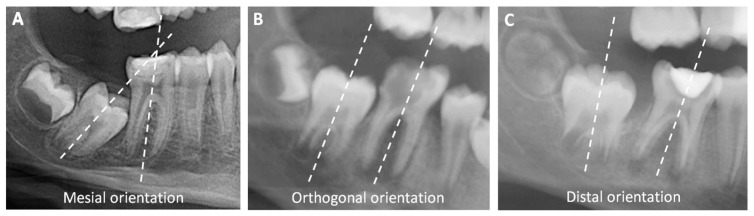
Orientations of mandibular SPM: (**A**) Mesial orientation, as the axes cross at the coronal part; (**B**) Orthogonal orientation, as the axes are parallel; (**C**) Distal orientation, as the axes cross at the apical part.

The author also collected the following data using a preoperative radiograph: the presence of TPM, the angulation of the SPM compared to the FPM (mesial, orthogonal, or distal) (Figure 1), and SPM Nolla’s stage.

For patients assessed one year after extraction, the author collected the following data: SPM movement (translation or version), the remaining space between the second permanent premolar (SPPM) and SPM (in millimeters), and whether any untreated decayed teeth were present

For patients seen one year after RCT, the author collected the following data: the presence of a sealed coronal reconstruction, the absence of clinical or radiological FPM infection, the need for endodontic retreatment or extraction, the FPM’s occlusal integrity, the radiological quality of the RCT, and the presence of other untreated decayed teeth.

For patients lost to follow-up before one year, the author collected the following data: duration of follow-up after treatment, reason for the first appointment at the hospital, and coronal reconstruction performed before loss to follow-up in cases of RCT.

### 2.5. Evolution Criteria Evaluated

#### 2.5.1. Extraction Group

For patients with extraction, the main criterion was the remaining space between SPPM and SPM at one year. A remaining space under 2 mm was considered a favorable evolution because it does not require specific orthodontic treatment to close the remaining space. This corresponds to levels one and two evaluated by Teo et al. [13]. A remaining space between 3 mm and 4 mm was considered less favorable, because orthodontic treatment is required to close the space. A remaining space over 5 mm was considered unfavorable, as it requires specific and more invasive orthodontic treatment, such as Temporary Anchorage Device (TAD).

#### 2.5.2. RCT Group

For patients with RCT, due to the lack of specific criteria for children, the favorable evolution criteria were based on those established by the European Society of Endodontology (ESE) for adults evaluated at one year [22]:

- Endodontic criteria: Absence of pain, swelling, and other symptoms; no sinus tract; and radiological evidence of a normal periodontal ligament space around the root.
- Functional criteria: No loss of function, when the FPM is in occlusion with antagonist FPM and there is a good contact with collateral teeth.

Both criteria are needed to assess the favorable evolution of RCT.

### 2.6. Statistical Analysis

The R software version 4.3.1 (The R Foundation for Statistical Computing platform) was used for the statistical analyses. For data comparisons including age data, a two-sample *t*-test was performed when the age distribution was normal, and a Wilcoxon rank-sum test when the age distribution was not normal. For all the other comparisons, a Pearson’s chi-squared test was used when the expected samples were greater than three, with a Yates’ continuity correction for expected samples between three and five. For expected samples under three, a Fisher’s exact test for count data was used. All analyses were performed with the assumption that the teeth were independent, even if some patients had several teeth treated.

## 3. Results

**Figure 2:**
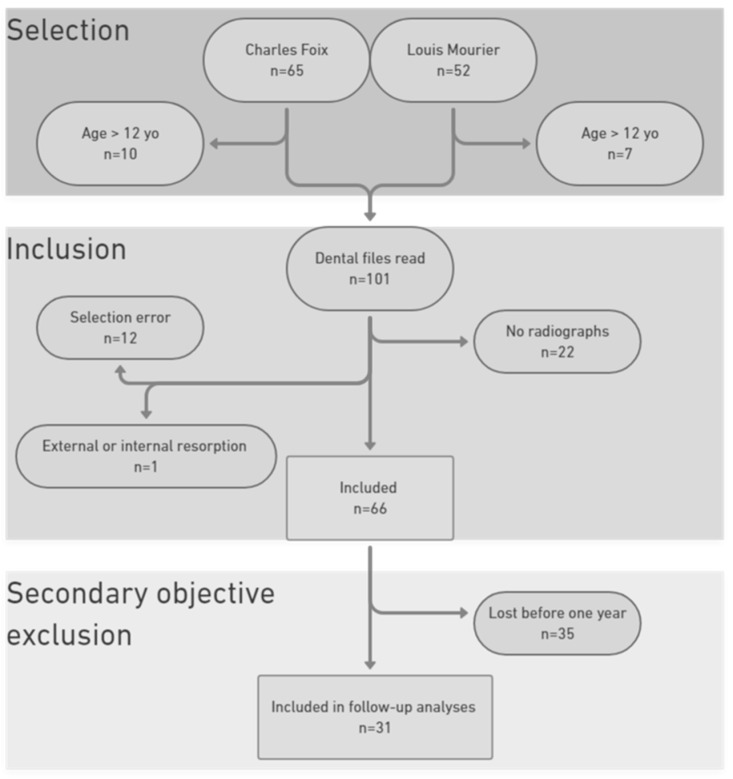
Flow chart of the entire study.

A total of 65 patients from Charles Foix Hospital (CFX) and 52 from Louis Mourier Hospital (LM) were found in the database. After applying the exclusion criteria, 39 patients at CFX and 26 at LM were included in the main objective analysis. A total of 31 of them were followed for one year or more and were included in the secondary objective analyses (Figure 2).

### 3.1. Population

**Figure 3:**
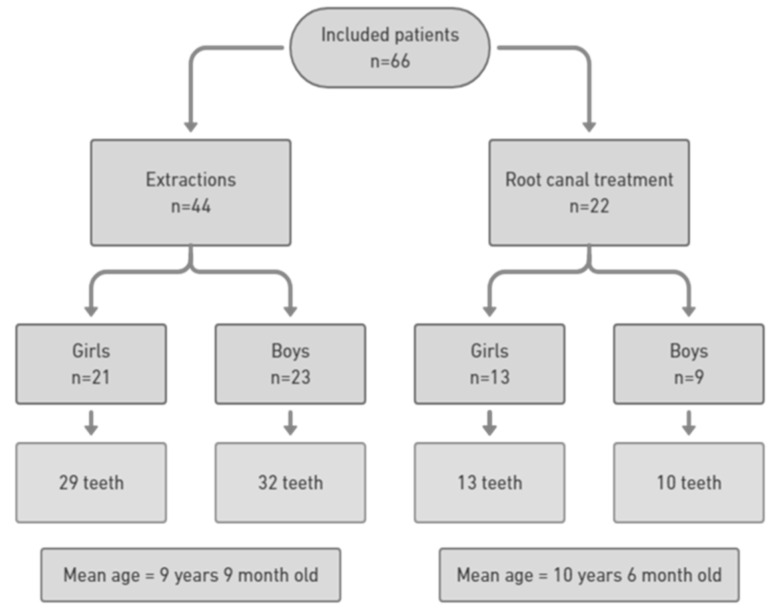
Sex, age, and teeth characteristics of the included patients.

A total of 66 patients were included in the study, representing 84 teeth. Of these, 44 patients had extractions, and 22 had RCT, representing 61 extracted teeth and 23 RCT (Figure 3).

The extraction and RCT groups presented a similar sex ratio (*p* = 0.46). However, the extraction group was slightly younger (normal age distribution in both groups, *p* < 0.05), with a mean age difference of around nine months.

### 3.2. Criteria Impacting the Therapeutic Decision (Table 1)

The two main criteria impacting the therapeutic decision were the presence of MIH (*p* < 0.05) and FPM decay affecting two tooth surfaces or more (*p* < 0.05). In these two cases, extraction was the preferred choice. These two criteria are not linked: teeth with MIH are not more decayed than other teeth (*p* = 0.96). Patients with MIH are therefore younger (normal age distribution, *p* < 0.001), but there are as many boys as girls with MIH (*p* = 0.82).

**Table 1:** Clinical and radiographic data at the treatment date.

Parameter		Extraction n = 61	RCT n = 23	*p*-Value
Arch	Maxillary	14	3	*p* = 0.48
	Mandibular	47	20	
Number of tooth surfaces decayed	Occlusal	6	10	*p* < 0.05
	2 surfaces or more	55	13	
MIH		29	2	*p* < 0.05
Presence of infection (AAP, abscess, cellulitis)		54	18	*p* = 0.40
SPM Nolla’s stage	5	4	-	*p* < 0.05
	6	5	6	
	7	27	1	
	8	19	7	
	9	2	-	
	Not reported *	4	9	
presence of TPM		44 (out of 52 assessed *)	7 (out of 10 assessed *)	*p* = 0.36
SPM angulation if mandibular	Mesial	5	1	*p* = 1
	Orthogonal	32	12	
	Distal	4	1	
	Not reported	6	6	

RCT, root canal treatment; MIH, Molar Incisor Hypomineralization; AAP, Acute Apical Periodontitis; SPM, second permanent molar; TPM, third permanent molar; * When the radiograph was not clear enough to assess this outcome, especially when there were only intra-oral radiographs, the tooth was removed from that analysis.

The decision was not influenced by the maxillary or mandibular position of the tooth (*p* = 0.48). The presence of an infection or the presence of TPM did not impact the decision either (*p* = 0.40 and *p* = 0.36 respectively).

The SPM Nolla’s stage was not always assessed before the decision and was not the main decision criterion. Indeed, nine RCT and four extractions were performed without knowing that criterion. On the other hand, all the other RCTs were performed when the SPM was between stages six and eight. Only four FPMs were extracted at SPM’s stage five, in two children treated under general anesthesia with non-restorable teeth. Two other FPMs were extracted at SPM Nolla’s stage nine, as they were root debris.

The SPM angulation was never assessed when the decision involved a mandibular FPM. For the study, we assessed to SPM angulation with radiographs, except for six RCTs and six extractions where the radiograph was not large enough.

### 3.3. Follow-Up Duration and Patients Lost to Follow-Up

A total of 48% of the patients were seen at one year (n = 31). This represents 41 treated teeth (Table 2).

The patients followed at one year were more often boys (*p* < 0.05) and more often had MIH (*p* = 0.03). The treatment type did not impact the follow-up (*p* = 0.28), nor did the age at the treatment date (non-normal age distribution, *p* = 0.44).

The follow-up appeared to be less well done in patients with larger carious lesions (defined as two or more decayed surfaces), although this difference did not reach statistical significance (*p* = 0.08) (Table 2).

An investigation into the reasons for loss to follow-up was conducted. Patients treated under general anesthesia (GA) or those attending appointments with endodontic specialists were not followed at the hospital, as they were referred by private practitioners. In these cases, the patient did not need to return to the hospital after treatment (n = 9). This is also the case for patients seen exclusively for emergency treatment due to dental pain, as they often faced challenges in securing appointments for comprehensive care (n = 10). The most concerning cases of lost follow-up involved patients who missed multiple appointments (n = 8) or did not schedule any subsequent visits following their comprehensive treatment (n = 8). These patients were primarily lost due to difficulties in establishing contact for new appointments, which accounted for the majority of the follow-up losses.

In the 14 endodontically treated teeth where patients were lost to follow-up, six were only restored with a temporary coronal restoration, such as Glass Ionomer Cement (GIC) (n = 5) or zinc oxide eugenol paste (n = 1).

### 3.4. Results of Extractions at One Year of Follow-Up

A total of 22 patients, representing 32 extracted teeth, were followed at one year (Table 3).

Among the seven maxillary teeth extracted, six had a favorable evolution. Only one tooth had a 6 mm space remaining between the SPPM and the SPM. The tooth was extracted when the SPM was at Nolla’s stage nine, and was root debris. The majority of SPMs translated (n = 5) or translated with a slight version (n = 1).

Among the 25 mandibular extracted teeth, only 10 of them had a favorable evolution. Four had a less favorable evolution, and eleven had an unfavorable evolution. A majority of the SPM movements were versions (n = 18). Regarding the SPM angulation before extraction, the mesially oriented SPMs (n = 3) had an unfavorable evolution, the orthogonally oriented SPMs (n = 20) had a majority of unfavorable evolution (n = 12), and the distally oriented SPMs (n = 2) had a favorable evolution. The two situations with the least favorable one-year evolution (9 mm of remaining space) both occurred when the FPM was extracted at Nolla’s stage five of the SPM.

No significant difference in favorable or unfavorable evolution was noted between maxillary and mandibular teeth (*p* = 0.09).

Furthermore, no significant difference in favorable or unfavorable evolution was noted whether the TPM was present (n = 24, including 11 favorable evolutions) or absent (n = 5, including three favorable evolutions) (*p* = 0.64).

Age at extraction also did not play a role in the evolution either (non-normal age distribution, *p* = 0.57)

The angulation of the SPM following space closure is also a key factor for long-term periodontal stability. In the maxillary SPM, the majority (n = 5) underwent translation during space closure, while only one exhibited version associated with the translation, resulting in a larger interdental space despite closure. That SPM was at Nolla’s stage eight at the time of FPM extraction, while the others were at stage five or six.

In the mandibular SPM, nine out of ten SPM with less than 2 mm of remaining space displayed version during space closure, with five of these cases involving both translation and angulation, and four cases showing only version. The only SPM with a complete translation was at Nolla’s stage eight at the time of FPM extraction. Among the 15 other mandibular SPM, only one with 3 mm of remaining space underwent both translation and version, while the others exhibited version without translation.

### 3.5. Results of Root Canal Treatments at One Year of Follow-Up

Nine patients representing nine teeth were followed at one year (Table 4). Among them, four teeth needed extraction or endodontic retreatment at one year due to carious recurrence, which was considered as an unfavorable outcome.

Among the two teeth with two decayed surfaces before treatment, one needed retreatment due to the recurrence of infection with an unsealed coronal reconstruction, and one required extraction due to carious recurrence. The other seven teeth had only occlusal decay before the treatment. Only two of them needed either retreatment (n = 1) or extraction (n = 1). There is therefore no statistical difference between teeth with occlusal decay and those with two-surface decay (*p* = 0.16).

As all the favorable outcomes were in cases of infected teeth (n = 5), which was the case in only two of the unfavorable outcomes (n = 4), the infectious status of the tooth was not a criterion for success (*p* = 0.16). The age at the time of treatment was not a criterion either (non-normal age distribution, *p* = 1).

### 3.6. Treatments Comparison at One Year of Follow-Up

To compare the two treatments, only the favorable evolutions were considered successful. This represents 16 of the 32 extractions, and five of the nine RCTs. No statistical difference was noted between the two treatments (*p* = 1).

The same result was obtained with the maxillary teeth, with six favorable evolutions out of seven extractions, and one RCT out of two (*p* = 0.42). Similarly, for the mandibular teeth, there were 10 favorable evolutions out of 25 extractions and four RCTs out of seven (*p* = 0.71).

Interestingly, regardless of the therapeutic strategy, more than 70% of patients still had active carious lesions on other teeth after one year (16 extractions out of 22 patients and seven RCTs out of nine patients). There was no statistical difference between the two treatments (72.72% of extractions versus 77.78% of RCTs, *p* = 1).

## 4. Discussion

The issue of appropriate treatment for necrosis of the FMP in children aged eight to twelve years is very important in our hospital departments, where we receive a significant number of children in this situation. This tooth is essential for chewing and growth, maintaining the vertical dimension of occlusion, and allowing the proper placement of other permanent teeth. It is also the first tooth to erupt, and therefore needs to have the longest lifespan on the arch. The question of its restoration is thus crucial. We have chosen to examine the various options when this FPM has one of the most unfavorable prognoses, that is, when it is necrotic. Limiting the therapeutic decision to the necrotic FPM is a first step toward a more comprehensive study evaluating the necessity of preserving or extracting severely damaged FPMs. To our knowledge, this retrospective study is the first to compare the different treatment options available in this situation within the same population.

Regarding the decision criteria for the two treatments, we could not identify all the criteria considered for the patients because the decision was rarely justified in the dental record. Therefore, we identified seven criteria frequently observed.

First, the presence of MIH seems to orient the dentist toward extraction, as 93.55% of teeth with MIH were extracted (*p* < 0.005). This criterion was also proposed by Ashley and Noar [23]. The difficulties in anesthetizing and achieving a sealed coronal restoration can explain why we prefer extraction in this case. The fact that the patients with MIH were younger in our cohort may also explain that decision, as it is more difficult to accept long treatment such as RCT at a young age.

Another criterion identified is the extent of the decay. Indeed, 90% of extractions were performed when the teeth had two or more surfaces decayed (*p* < 0.05). This factor seems to be an RCT prognosis factor as well, as all the RCT performed when two surfaces were decayed had an unfavorable evolution at one year. This aligns with the conclusions of Bernardo et al. [24], conducted between 2000 and 2007 on 892 composites performed on premolars or molars between eight and twelve years old. The seven-year survival rate is 93% when the restoration is only occlusal, dropping to 80% for two surfaces, and then to 60% for three or more surfaces restored. This study was carried out with experienced practitioners and annual follow-up of all patients, which may explain the good results for two-surface restorations compared to the results presented here. It is therefore necessary to anticipate the difficulties of coronal restoration before making the therapeutic decision.

The SPM Nolla’s stage was also identified as an important criterion for extraction. Indeed, the FPM extracted at SPM’s stage nine and the two FPMs extracted at SPM’s stage five were the teeth with the least favorable evolution at one year. This confirms the results of Teo et al. and Ertugrul et al., where the earlier the teeth were extracted in the development of SPM, the greater the risk of persistence of the space at five years, up to stage nine where almost no spontaneous space closure was observed [11,25]. We therefore confirm the recommendations of Cobourne et al. and Ashley and Noar to consider extraction only when the SPM is between Nolla’s stages six and eight [23,26].

Contrariwise, the presence of an infection, such as AAP, abscess, or cellulitis, seems not to be an important criterion for RCT success. Indeed, all the RCT which evolved favorably at one year had an infection before treatment (*p* = 0.1667). This confirms the results from Dhafar et al., where 77.9% of successful RCT were performed with periapical lesion at treatment time, versus 60.3% of the unsuccessful RCT [17]. Therefore, this criterion does not need to be considered when the child does not have a medical condition requiring comprehensive medical treatments.

For the mandibular teeth, the SPM angulation seems not to be an important criterion either. Indeed, all the mesial angulations had an unfavorable evolution at one year, whereas all the distal angulations had a favorable evolution. These results contrast with those of Teo et al. and Patel et al., the only other team assessing that outcome [10,13]. The difference could be due to the difference in follow-up duration, as they performed a five-year follow-up. We therefore confirm the results of Nordeen et al., who assessed the same criterion in 168 mandibular quadrants, and did not measure any significant difference in result depending on the SPM angulation [27]. That criterion needs to be assessed in larger groups before being taken into account for the therapeutic decision.

Similarly, the presence of the TPM was not a criterion for favorable evolution in the extraction group. Only 44% of the extractions performed when the TPM was present had a favorable evolution at one year (*p* = 0.6424). These results contrast with those from Teo et al. on mandibular teeth, where only one favorable evolution among 33 teeth was observed when the TPM was absent [13]. This difference could be due to the difference in follow-up duration, as the patients we had were younger at the follow-up than the patients from Teo et al. [13]. Our patients were therefore too young to have a mature TPM impacting the SPM mesialization. A longer follow-up would be necessary to assess that outcome, which is nearly impossible with our patients, considering the loss of follow-up at one year. The absence of TPM is in favor of the FPM’s RCT, as only one molar will be maintained on the arch, creating long-term occlusal and functional disorders. Contrariwise, the presence of TPM could be in favor of the FPM extraction, as it may improve future TPM eruption and prevent pathology associated with impaction [28].

The arch concerned was not a decision criterion in our cohort either (*p* = 0.48). That element could be part of the decision, as the spontaneous space closure is more predictable after maxillary FPM extraction. In our cohort, only one maxillary SPM (14.3%) had an unfavorable evolution, and it was at Nolla’s stage nine when the FPM extraction was performed. Teo et al. also had 92% of favorable evolution at the maxillary teeth, and Patel et al. had 89.9% [10,11]. This is not the case for the mandibular arch, as we had only 40% of favorable evolutions, confirming the five-year results of Teo et al. (66%) and Patel et al. (49%) [10,11]. The maxillary RCT is also more complex because of the need to work in indirect vision and the maxillary FPM’s complex anatomy.

Moreover, even if the space closure is complete, a version of the SPM during the space closure could lead to a larger interdental space, undermining the long-term periodontal stability [29]. In our cohort, the majority of maxillary SPMs which performed space closure translated without version, but it is not the case for the mandibular SPMs, with only one translation out of ten. Even if we could not perform statistical analyses due to the small sample size, this confirms the results of Bakkal et al., with a more favorable evolution at the maxilla [29]. Demir et al. also noted the heterogeneous situation at the mandibular arch that we observed in our cohort, but also reported that 80% of the patients did not notice that problem [30]. We could not have a quality-of-life assessment in our cohort to compare with their results, as it was not asked at the one-year post-extraction appointment.

In cases of unfavorable evolution, orthodontic treatment could be a good solution to close the space with a favorable interdental space. We could not evaluate the increased need of orthodontic treatment after FPM extraction in our cohort, as an orthodontic assessment was not performed before for the majority of cases. That increased need of orthodontic treatment could be around 25% of the cases, especially for the mandibular extractions [31].

While our findings provide valuable insights into the relationship between extractions and root canal treatments (RCTs) in children, several limitations should be considered. First, the sample size was relatively small, which may limit the generalizability of the results. Additionally, the use of 2D imaging techniques may compromise the accuracy in evaluating the outcome of both treatments, due to the inherent limitations in precision associated with 2D radiographs. As this study was conducted using existing dental records from the AP-HP, it was not possible to obtain additional radiographs specifically for the purposes of this research. Consequently, we relied on routine radiographs, as is common practice in similar studies by other authors [10,11,13,15,23,25,26]. Finally, a comparison between operators for the RCTs could not be performed due to the small sample size and the variability in operator experience (including endodontic specialists, dental residents, and students). Although we evaluated the radiographic quality of the RCTs, which was satisfactory in all cases, this alone is insufficient to fully assess the quality of the RCTs. Factors such as the treatment protocol used and the anatomical characteristics of the teeth should be evaluated in larger studies, as this was not feasible within our cohort. These limitations suggest that further research with larger, more diverse populations and controlled methodologies is necessary to strengthen the validity of these findings.

This study highlights the difficulty we have in the French public pediatric dentistry departments in monitoring patients for more than six months. Indeed, more than half of the patients were lost to follow-up before one year, and we could not contact them again to continue the follow-up. This may have been due to several causes: (1) The inclusion period corresponds to the COVID-19 crisis, with the lockdown creating a sudden stop in treatments and scheduled follow-ups, and difficulties in restarting treatments after the lockdown. (2) The administrative constraints of hospital follow-up are important, particularly with longer waiting times, forcing the parents to take holidays to bring their child at the appointments, and frequent changes in practitioners.

Owing to the study’s retrospective design, it was not feasible to ascertain the oral health literacy level of the parents. Indeed, it is not a commonly evaluated at the first appointment at the hospital. This parameter could explain some of the loss to follow-up in patients with socio-economic difficulties, who are more often treated at the hospital [32]. Due to language barriers and a higher need for treatments with longer appointment times, which are difficult to manage when having precarious work, these patients may have more difficulties keeping their appointments. Patients with lower income may also have more decayed FPMs at a young age, as the oral health literacy in that population is poorer [33].

This study also highlights another difficulty faced by public pediatric dentistry departments, which is the high risk of caries. Only eight of the thirty-one patients seen at the one-year appointment were free of cavities. This underscores the challenges in controlling the risk of caries in this population. Indeed, children with decayed FPMs are known to have a higher DMFT/dmft than the other children, increasing the therapeutic challenge of these FPMs [34].

## 5. Conclusions

Our findings underscore the importance of carefully evaluating each case of FPM necrosis in children, considering both extractions and RCT as viable options based on individual patient characteristics. Given the study’s limitations, including the small sample size, further research with larger cohorts and standardized treatment protocols are needed. Clinically, it is crucial to perform a comprehensive examination to assess five key criteria before making a treatment decision: (1) Is it possible to perform a long-term sealed coronal reconstruction to swiftly ensure an endo-prosthetic continuum? The sealing criterion is essential to assess before performing the RCT, as it will be the main criterion of long-term therapeutic success [35]. (2) What is the SPM Nolla’s stage? The extraction must be performed only between stages six and eight. This parameter will be important for the extraction decision. (3) What are the follow-up possibilities? It is an important question to discuss with parents, as both decisions will require different follow-up. RCT will require many treatment appointments and close monitoring of the coronal reconstruction, and extraction may need orthodontic treatment in case of unfavorable evolution. (4) Which arch is concerned? The extraction evolution is more favorable in the maxillary arch. (5) Is the TPM present? It is a long-term occlusal and functional stability criterion in case of extraction.

All these factors must be considered before making a decision, and orthodontic advice can be valuable in the most uncertain cases.

## Figures and Tables

**Table 2:** Comparative characteristics of patients seen at one year and patients lost on follow-up, in number of teeth.

		Seen at One Year n = 41	Lost on Follow-Up n = 43	*p*-Value
Sex	Boys	27	15	*p* < 0.05
	Girls	14	28	
MIH		20	11	*p* = 0.03
Treatment	Extraction	32	11	*p* = 0.28
	RCT	9	29	
Number of tooth surfaces decayed	Occlusal	11	5	*p* = 0.08
	2 surfaces or more	30	38	
Age at treatment date		9 years 10 months	10 years	*p* = 0.44

MIH, Molar Incisor Hypomineralization; RCT, root canal treatment.

**Table 3:** Comparative characteristics of maxillary and mandibular teeth one year after first permanent molar extraction.

		Maxillary n = 7	Mandibular n = 25	*p*-Value
SPM movement	Translation	5	1	
	Version	1	18	*p* < 0.001
	Translation-version	1	6	
Remaining space between SPPM and SPM	0–2 mm	6	10	
	3–4 mm	-	4	*p* = 0.09
	5–9 mm	1	11	
Other untreated decayed teeth at one year		6	17	*p* < 0.05

SPM, second permanent molar; SPPM, second permanent premolar.

**Table 4:** Comparative characteristics of maxillary and mandibular teeth one year after FPM endodontic treatment.

		Maxillary n = 2	Mandibular n = 7
FPM’s occlusal integrity		1	6
FPM sealed coronary reconstruction		1	4
Good radiological quality of RCT		2	7
Absence of clinical or radiological FPM infection		1	5
FPM treatment need	Retreatment	-	2
	Extraction	1	1
Other untreated decayed teeth at one year		1	6

FPM, first permanent molar; RCT, root canal treatment.

## Data Availability

The raw data supporting the conclusions of this article will be made available by the authors upon request at the corresponding author.
